# Role of stimulus dose on neuropsychological functioning after electroconvulsive therapy in patients with major depressive disorder

**DOI:** 10.3389/fpsyt.2024.1443270

**Published:** 2024-09-26

**Authors:** Lea Rummel, Katharina Göke, Alexandra Philipsen, René Hurlemann, Maximilian Kiebs

**Affiliations:** ^1^ Department of Psychiatry and Psychotherapy, School of Medicine & Health Sciences, University Hospital Oldenburg, Oldenburg, Germany; ^2^ Department of Psychiatry and Psychotherapy, University Hospital Bonn, Bonn, Germany; ^3^ Institute of Medical Science, University of Toronto, Toronto, ON, Canada

**Keywords:** electroconvulsive therapy, neuropsychology, treatment dose, charge, depression, cognition

## Abstract

**Introduction:**

Electroconvulsive therapy (ECT) is the most effective treatment for patients suffering from treatment-resistant depression but its use is often limited by the concern for cognitive side effects. This study examines the effect of ECT on autobiographical and verbal memory compared to a healthy control group and the impact of the mean stimulus dose on cognition after ECT.

**Methods:**

Autobiographical and verbal memory were assessed in depressed patients and healthy controls before the first and within one week after the last ECT treatment. Neuropsychological testing included the Autobiographical Memory Interview, the Verbal Learning and Memory Test and five tests from the Cambridge Neuropsychological Test Automated Battery. The mean charge delivered across the ECT series and the total number of sessions were examined in relationship to cognitive impairment after ECT using a multiple regression model.

**Results:**

Autobiographical memory was significantly impaired after ECT treatment compared to healthy controls. Baseline scores were lower for depressed patients on all cognitive domains. Improvements in performance after ECT were found on tests for executive functions and working memory. Effects of the mean charge delivered on cognitive functioning after ECT were heterogeneous across cognitive domains but significant for verbal retrograde memory.

**Conclusion:**

ECT led to autobiographical memory impairment. The relationship between mean charge delivered and cognitive performance is heterogeneous across different cognitive domains and requires further research. Significant effects of the mean charge delivered were found without a significant difference in cognitive functioning compared to a healthy control group.

## 1 Introduction

Electroconvulsive therapy (ECT) is currently the most effective treatment for treatment-refractory depression (TRD). In comparison to pharmacotherapy or other forms of brain stimulation, ECT yields higher response and remission rates, even in more severely ill patients (1–4). ECT involves an (ultra-) brief electrical current being passed through the brain via electrodes to induce a generalized seizure while the patient is under general anesthesia. Despite its long history, the underlying mechanism of antidepressant action of ECT remains unclear and is still under investigation (5, 6).

Although haven itself proven safe for patients across the adult life span including elderly patients (7, 8), as well as tolerable and highly efficient for TRD, ECT is still associated with stigma (9–11).

Adverse effects during or right after a treatment session include nausea, headaches, muscle or jaw pain (12–14). However, part of the stigma is due to the undesirable cognitive effects associated with the treatment (9, 15). Whereas some authors claim that ECT does not cause significant neuropsychological impairment, which is more likely to be a depressive phenomenon (16), others are more cautious (17, 18).

As one of the most critical cognitive adverse effects, several studies showed a significant decline of retrograde autobiographical memory immediately after ECT treatment and up to six months after the last treatment (17, 19–21). Additionally, other neuropsychological domains that might be affected by ECT treatment include processing speed, attention, verbal fluency, visual memory and executive functions (22–24).

Verwijk et al. found that ECT results in a loss of autobiographical memory and impairment of verbal fluency, anterograde verbal and non-verbal memory immediately after brief pulse right unilateral RUL-ECT. A reduction of processing speed as well as an impairment of working memory were found to a lesser extent (21, 24). Subjectively, memory worsening following ECT was found to be reported only by a minority of patients (25). Often, subjective memory complaints are strongly correlated with depression severity, rather than objective cognitive impairment and improve after ECT treatment (26–29). Nuninga et al. (30) also found transient adverse cognitive effects for verbal memory and learning as well as verbal fluency following bilateral ECT, but no persisting impairments. However, it has been shown that ECT can cause a significant impairment of autobiographical memory persisting up to three months after the procedure (20, 31). Semkovska and McLoughlin (23) found that cognitive abnormalities associated with ECT are mainly limited to the first three days posttreatment, and some domains, including processing speed and working memory, showed improvement 15 days post ECT. For visual and visuospatial memory, significant impairments during and within one week after ECT were found, which mostly resolved when testing one month after the last ECT treatment (22).

In light of these heterogeneous findings, predicting the occurrence and understanding the origin and nature of the neurocognitive effects of ECT remain a challenge. Apart from individual patient characteristics, bilateral electrode placement as well as longer pulse-width predict stronger cognitive impairment after ECT (29, 32–37). Furthermore, increased frequency of ECT treatments was also associated with more cognitive side effects (38, 39). Regarding the impact of electrical dose, studies have found that a higher dosage relative to individual seizure threshold predicted stronger cognitive side effects rather than the absolute electrical dose administered, however, the antidepressant effect of ECT also increased with dosage (40–45). For instance, fixed high dose stimulation was associated with reduced autobiographical memory and longer time to reorientation compared to titrated moderately suprathreshold stimulation (40). Sackeim et al. showed that RUL high dosage stimulation was twice as effective as low dose stimulation (34). More recent studies showed that lowered ECT stimulus doses were associated with less subjective memory worsening and better verbal learning without compromising efficacy, but this association was only detectable up to three days after the final ECT treatment (25, 46).

This study examines the effects of ECT on autobiographical and verbal memory in comparison to a healthy control group. Additionally, the study aims to determine the extent to which cognitive impairment can be predicted by the mean electrical charge delivered across the ECT series and the total number of ECT sessions. Firstly, it was hypothesized that there would be significant differences in autobiographical and delayed verbal memory tests between depressed patients and healthy controls and that memory performance scores decrease following ECT treatment. Additional cognitive domains were included for exploratory purposes. Secondly, it was hypothesized that a higher mean stimulus energy, measured in milli Coulomb (mC) and calculated as mean charge across the ECT series, rather than the total number of ECT sessions, would be associated with greater cognitive impairment following an ECT series.

## 2 Materials and methods

### 2.1 Subjects

Data were collected from adult patients who received ECT treatment from January 2018 to December 2019 at the University Hospital Bonn (clinicaltrials.gov: NCT03490149). The indication for ECT treatment was made by the treating psychiatrist for patients with a clinical diagnosis of a unipolar or bipolar depressive disorder and who failed to respond to treatment with at least two antidepressant medications. Patients were excluded from the study in case of heart disease, certain neurological conditions, diagnosed hearing loss, thyroid dysfunction, prior treatment with at least one ECT within the last three months and a history of treatment with deep brain stimulation. In terms of psychiatric disorders, patients with the following diagnoses were excluded: secondary or substance-induced depression, psychotic disorders, generalized anxiety disorder, panic disorder or social phobia.

The sample of this study consisted of 21 patients and 19 control participants, matched to the therapy group in terms of gender and age. All participants gave written, informed consent to take part in the study.

### 2.2 ECT

Brief pulse ECT treatments were administered twice weekly with a constant current apparatus (Thymatron IV). For anesthesia, propofol (1-2 mg/kg) and succinylcholine (1 mg/kg) were used. Prior to treatment, all patients received positive pressure ventilation with 100% oxygen. Seizure threshold was determined individually during the first ECT session for all patients (44), and the therapeutic dosage was set to at least four times initial seizure threshold. In case of insufficient seizures, the energy was raised accordingly and the stimulation was repeated, with a maximum of three stimulations in a single session. All treatment related data was collected using the longitudinal data collection tool GENET-GPD (47).

### 2.3 Neuropsychological testing

All neuropsychological tests were administered by trained personnel before starting an ECT series and within one week after finishing the series. Clinical improvement was assessed using the 21-item Hamilton Rating Scale for Depression (HAMD) (48).

The following tests were used in order to evaluate different aspects of cognitive functioning after ECT treatment: Verbal Learning and Memory Test (VLMT), Autobiographical Memory Interview – Short Form (AMI-SF). Exploratively, five tests from the Cambridge Neuropsychological Test Automated Battery (CANTAB) were used for assessment of other cognitive domains.

#### 2.3.1 VLMT

The VLMT is based on the Auditory Verbal Learning Test (AVLT), originally developed by Rey (49, 50). This standardized German version is frequently used for the assessment of verbal declarative memory (51). Subjects are read a list of 15 words on five successive trials with a free-recall task after each trial. Next, a distraction list is presented as interference with consecutive recall. The subjects are then asked to recall as many words as possible from the original list. This is repeated after 30 minutes. The primary variables of interest were the VLMT total score (recall sum across five successive trials), and the VLMT delay score (free recall after 30 min. delay).

#### 2.3.2 AMI

The AMI-SF (52) quantifies the extent of retrograde amnesia for autobiographical events following an ECT course. The test consists of six different parts, each covering different aspects of autobiographical memory (family member, travel, New Years, birthday, employment, physical illness). Amnesia scores are calculated by dividing the post-treatment scores by the baseline scores and multiplying the result by 100 in order to obtain a percentage.

#### 2.3.3 CANTAB

Exploratively, five different tests were chosen from CANTAB, which provides a rapid and non-invasive method of cognitive assessment that is increasingly used in examining cognitive effects after ECT (22, 53, 54). Advantages of the CANTAB tests are their efficiency, their highly standardized and digital administration and the automated response recording with millisecond precision (55). Below, each test used is summarized briefly.

- Delayed Matching to Sample (DMS) is a test for visual memory. The subject is shown a visual pattern made up of four sub-elements. Simultaneously or after a brief delay (4 or 12 s), four choice patterns are presented on a screen and the subject is instructed to touch the pattern that matches the sample previously shown. The subject is given a total correct score, expressed as a percentage.
- One Touch Stockings of Cambridge (OTS) assesses executive function, working memory and planning. The subject is presented with two displays containing three colored balls. The balls in the lower display must be moved one at a time in order to copy the pattern shown in the upper display with increasing complexity. The subject is given a score, representing the mean number of choices needed for the correct pattern.
- Rapid Visual Information Processing (RVP) is a test of visual sustained attention. The subjects are presented with digits from two to nine, appearing in a box on the computer screen at the rate of 100 digits per minute. Subjects are requested to detect a target sequence of three digits. The subject is given a total score (total hits).
- Spatial Working Memory (SWM) tests the subject’s ability to retain spatial information and to manipulate remembered items in working memory. The subject is shown a number of colored boxes, in which the subject should find one blue “token”. The number of boxes presented on the screen is gradually increased from three to eight boxes, as well as changing color and position of the boxes. The subjects must touch each box until one opens with a blue token inside. This is repeated for the next blue token. An error occurs when touching a box in which a blue token has already been found. The subject is given a total error score.
- Pattern Recognition Memory (PRM) is a test of visual pattern recognition memory in a two-choice forced discrimination paradigm. Firstly, the subject is presented with a series of 12 colored visual patterns, each pattern presented for three seconds. In the following recognition phase, the subject must choose between a pattern they have already seen and a novel pattern. The score for each subject is expressed as a total correct score.

### 2.4 Statistical analysis

To analyze cognitive impairment following a series of RUL-ECT in comparison to a healthy control group, a linear mixed model was used for each neuropsychological test including the variables timepoint, group and age as main effects. Effects of timepoint, group and their interaction were examined. The distribution of variances was assessed visually. In order to examine the association between stimulus energy across ECT treatment and cognitive performance, the mean charge delivered across all ECT sessions in the series was calculated for each patient. Mean charge delivered, number of ECT sessions and age were included as independent variables in a multiple regression model, with absolute change scores of cognitive measures as dependent variable. Pearson correlations were calculated respectively. All raw scores were z-transformed prior to analysis, which was performed in R statistics 4.2.3 (56). Multiple testing was controlled for via the false-discovery rate using the method of Benjamini and Hochberg (57).

## 3 Results

### 3.1 Participant characteristics

Demographics and clinical characteristics are reported in **Table 1**. Depressed patients and healthy controls showed no significant differences regarding sex, age and education. Depressed patients had a significant higher body mass index (p = 0.016).

**Table 1:** Participant Characteristics.

Variable	N	Patients, N = 21^1^	Controls, N = 19^1^	p-value^2^
**Sex**	40			0.5
m		9 (43%)	10 (53%)	
w		12 (57%)	9 (47%)	
**Age**	40	48 (37, 56)	43 (33, 56)	0.5
**BMI**	36	30 (24, 34)	24 (22, 26)	0.016
**Education** ^3^	36			0.5
2		0 (0%)	1 (5.3%)	
3		3 (18%)	3 (16%)	
4		2 (12%)	0 (0%)	
5		7 (41%)	5 (26%)	
6		0 (0%)	2 (11%)	
7		5 (29%)	7 (37%)	
8		0 (0%)	1 (5.3%)	
**Diagnosis**	40			
F31.4		5 (23.8%)	0 (0%)	
F32.2		1 (4.8%)	0 (0%)	
F33.2		14 (66.6%)	0 (0%)	
F33.3		1 (4.8%)	0 (0%)	
NA		0 (0%)	19 (100%)	
**Duration of depressive episode [month]**	17	13 (9, 22)	NA	
**Number of depressive episodes**	36	7.0 (3.0, 10.0)	0.0 (0.0, 0.0)	
**Age at first treatment**	18	30 (22, 40)	NA	
**HAMD total score**	40	20 (17, 22)	0 (0, 2)	
**Mean charge delivered [mC]**	21	330.69 (232.27, 421.10)	NA	
**Number of ECT sessions**	21	11.86 (10, 13)	NA	

^1^n (%); Median (IQR).

^2^Pearson’s Chi-squared test; Wilcoxon rank sum test; Fisher’s exact test.

^3^2 = grade 7-12 (without graduating high school); 3 = graduated high school; 4 = part college or university; 5 = graduated 2-year college (Associates Degree); 6 = graduated 4-year college (Bachelor Degree); 7 = part graduate or professional school; 8 = completed graduate or professional school.

### 3.2 Efficacy of ECT treatment

Efficacy of the ECT treatment was assessed by comparing the mean HAMD score before and after ECT treatment. The mean HAMD score decreased significantly after a course of ECT from 19.73 ± 4.05 to 6.6 ± 4.37 (p < 0.01).

### 3.3 Longitudinal effects patients vs. controls

#### 3.3.1 AMI and VLMT

**Figure 1:**
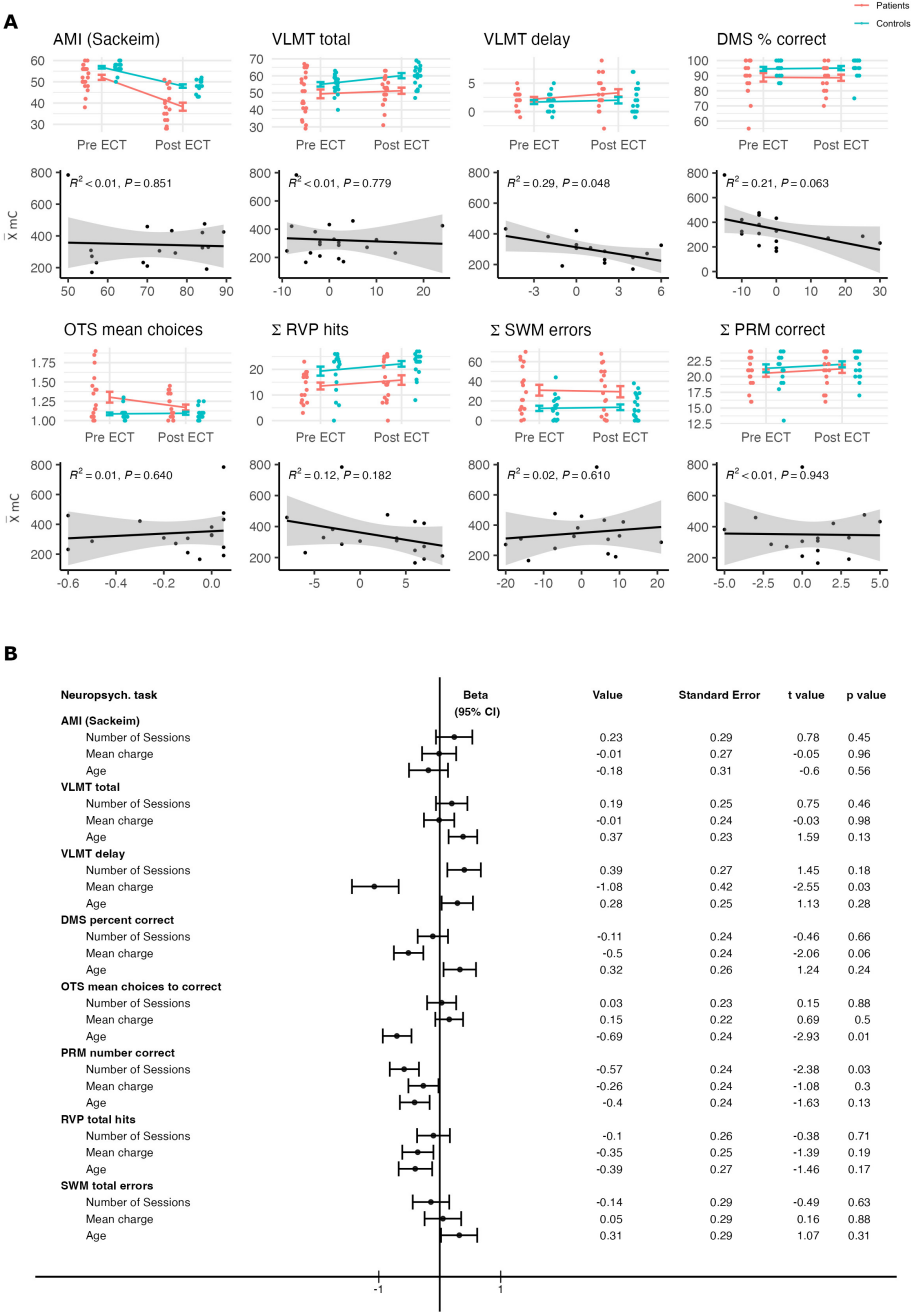
**(A)** Above, comparison between patient and control group for performance on each neuropsychological test before and after ECT treatment. Below, Pearson´s correlational analyses between the mean charge delivered (mC) and the change in performance from before to after ECT treatment on each neuropsychological test and their respective p values. **(B)** Results from the multiple regression model for each neuropsychological test conducted in the analysis. Data were z-transformed prior to the analysis. Components of the regression model include the mean charge delivered across the ECT series (mC), age and total number of ECT sessions. Beta value (including the 95% confidence interval), standard error, t value and p value were added for each factor respectively. Beta values were pseudo-log transformed for visualization purposes using the ggallin package (58).

In comparison to healthy participants, depressed patients scored significantly lower on the AMI-SF at both timepoints (p < 0.01) and the scores also decreased significantly after ECT treatment compared to before treatment (p < 0.01; see **Figure 1**). The mixed model found a significant interaction effect between group and timepoint (p < 0.01) and no significant effect of age on the AMI score (p = 0.53).

For the VLMT variables, the linear mixed model found a significant difference in performance between patients and healthy control participants for all tested variables (all p < 0.05) as well as a significant effect of the timepoint for VLMT total score (p < 0.01). No significant interaction effect was found for either VLMT variable (all p > 0.01). Performance on the VLMT was negatively influenced by the age of the participants (all p < 0.01).

#### 3.3.2 CANTAB

In the DMS task, depressed patients scored significantly lower than healthy controls (p = 0.01). However, there was no significant difference between initial baseline scores and subsequent tests after ECT treatment (p = 0.93) and no significant interaction effect was found between group and timepoint (p = 0.8) as well as no age effect (p = 0.44).

In the OTS task, depressed patients needed significantly more choices for the correct result in comparison to healthy control participants (p < 0.01), but their scores improved significantly after ECT (p = 0.03). The mixed model found a significant interaction effect between group and timepoint (p = 0.01). However, the model also found a significant effect for age (p = 0.01).

For visual processing, the depressed group had significantly fewer hits in total compared to the healthy group (p < 0.01). The linear mixed model found a significant difference in total scores before and after ECT series (p < 0.01), also indicating an improvement after the ECT series. There was no significant interaction effect between group and timepoint detectable in the model (p = 0.78).

In the SWM task, depressed patients made significantly more errors in total compared to the healthy control group (p < 0.01), but there was no significant difference before and after ECT series (p = 0.79). There was also no significant interaction effect between group and timepoint detectable in the model (p = 0.74). However, the linear mixed model found a significant negative impact of the participants´ age on the performance on the SWM task (p < 0.01).

For the PRM task, there were no significant effects for group or timepoint as well as no interaction effect or age effect detectable (all p > 0.01).

### 3.4 Role of treatment charge

#### 3.4.1 AMI and VLMT

The mean charge delivered across all patients was 330.69 mC (SD = 140.47) as reported in **Table 1**. On average, the patients received a mean total of 11.86 ECT sessions (SD = 2.15). For the AMI score, including a total of 17 patients in the analysis due to incomplete post-treatment testing, the multiple regression model found no significant effect of the mean charge on the AMI score (p = 0.96). A significant negative effect of the mean charge delivered in the ECT series on verbal memory was found for the VLMT delay score (p = 0.03), but not for the VLMT total score (p = 0.98). Due to missing follow-up data, only 14 patients could be included in the analysis for the VLMT delay score, whereas 20 patients were included for the VLMT total score. Age and the number of ECT sessions had no significant impact on either variable (p > 0.1).

#### 3.4.2 CANTAB

Exploratively, multiple regression models were also calculated for the five CANTAB variables. A higher mean charge influenced the performance on the DMS test, with lower overall percent correct in comparison to lower mean charge, although this trend was not significant (p = 0.06). A multiple regression model calculated for the total errors in the DMS task found a significant effect of mean charge (p = 0.01), indicating that a higher mean charge was associated with more errors on the task. Age and the number of ECT sessions had no significant impact on cognitive performance (p > 0.1). For the OTS task, a higher mean charge had no significant impact on the mean choices to correct score (p = 0.5). As already mentioned in the ANOVA analysis, the model detected a significant influence of age on the test score (p = 0.01). The multiple regression model also found a significant impact of the mean charge on the mean latency to first choice score (p = 0.04). In the RVP task, the model did not find a significant impact of the mean charge on the total hits in the task (p = 0.19), but the total misses score was found to be significantly influenced by the mean charge delivered over the ECT course with more misses when a higher mean charge had been applied (p = 0.02). The number of ECT sessions had no significant impact on either variable (p > 0.1). No significant effects were found for the SWM task, indicating that performance on the SWM task was significantly influenced by neither the mean charge delivered nor the number of ECT sessions (p > 0.1). A significant effect for the number of sessions was found for the PRM task (p = 0.03), whereas no significant effect was detected for the mean charge delivered (p = 0.3). Age had no significant impact on performance in the PRM task (p > 0.1). Because not every CANTAB test was completed by each patient, four patients were not included in the analysis of the DMS, OTS and RVP tasks and five patients in the analysis of the SWM and PRM tasks, respectively. After correcting for multiple testing, using the method of Benjamini and Hochberg (57), no significant results were found anymore.

## 4 Discussion

This study examined cognitive performance on autobiographical and verbal memory tests before and after ECT treatment compared to a healthy control group. As expected, ECT did lead to short-term impairments in autobiographical memory compared to healthy controls. A significant decline in AMI scores before and after ECT treatment compared to a healthy control group was shown. These results are supported by a number of studies that also found autobiographical memory impairment shortly after ECT treatment (19–21, 30). Moreover, depressed patients had significantly lower AMI scores than healthy controls across both timepoints, indicating that major depressive disorder (MDD) is associated with memory dysfunction, which is also in line with previous research (59–61). Contrary to our expectations, this study found no verbal memory impairments after ECT treatment. Still, baseline scores were significantly lower in the depressed group. In a similar study, Verwijk et al. found transiently disrupted verbal memory immediately after brief-pulse ECT (21). Biedermann et al. even found significant improvement of verbal memory after ECT treatment (62).

To explore other cognitive domains and their dependency on ECT treatment, five CANTAB tests were included in the exploratory analysis. Those tests focused on executive functions and planning, visual information processing, spatial working memory and visual pattern recognition memory. For all tests, except the PRM task, baseline scores were significantly lower in the depressed group. This is in line with previous research showing moderately impaired memory as well as executive functions and working memory for depressed patients (59, 60, 63). Tests for working memory and executive functions may not be impaired by ECT treatment, but rather dependent on the age of the participants and performance even improved significantly after ECT treatment for the OTS and RVP task (23, 64).

Although it is widely established that ECT causes significant cognitive side effects, there are remaining questions on how these cognitive side effects are influenced by technical ECT parameters. It is known that ultra-brief pulse ECT as well as unilateral ECT are associated with fewer cognitive side effects, but no or only a slight decline in efficacy compared to brief-pulse or bilateral ECT (4, 29, 35, 43, 65–67). This study focused on the impact of the mean charge delivered across the RUL-ECT series as well as the total number of RUL-ECT sessions on autobiographical and verbal memory function after RUL-ECT treatment. In the literature, it has been described that a fixed high dose stimulation (403 mC) was associated with impaired autobiographical memory and longer time to reorientation, compared to titrated moderately suprathreshold (2.25 x) stimulation, but with higher efficacy (40). Not the absolute electrical dosage but rather the degree to which dosage exceeds threshold is related to the magnitude of acute cognitive impairments after ECT (42).

The results from this study, using the empiric titration method with RUL-ECT at least four times initial seizure threshold, implicate that a higher mean charge delivered across an ECT series may in fact predict stronger cognitive side effects, but these findings are heterogeneous across different cognitive domains. As expected, a higher mean charge across the RUL-ECT series predicted a lower VLMT delay score, but not a lower VLMT total score or lower AMI score. Moreover, a higher mean charge was associated with lower scores on the DMS task as well as on the RVP task, although these trends were not significant. In line with results from Kirov et al., the number of previous ECT sessions had no significant impact on cognitive deficits after ECT, with the exception of the PRM task (68).

This study highlights the importance of interpreting studies cautiously when they lack a healthy control group. Significant effects of the mean charge delivered on cognitive performance were found even when there was no significant difference in cognitive functioning compared to a healthy control group, which for instance applies to the VLMT delay score.

Due to the small sample size, the reported results have to be considered as rather preliminary but they serve as a guide for future studies with larger sample sizes focusing on the how the stimulus dose might predict cognitive performance after ECT.

In clinical settings, monitoring specific stimulation parameters in combination with potential cognitive side effects after ECT treatment might be useful. Future research should continue with predicting side effects after ECT in different cognitive domains based on different technical parameters. More research is needed to distinguish specific cognitive impairments following ECT from depressive phenomena and age-related decline in cognitive functioning. Understanding the nature of the cognitive side effects after ECT and looking for specific predictors is essential in further improving ECT practice and in diminishing residual stigma.

### 4.1 Limitations

The results of this study are limited by the sample size and the large number of tests that were assessed for each participant, although some were added for explorative reasons only. Furthermore, neuropsychological testing was conducted within the week after completion of the RUL-ECT series. This study is not able to differentiate whether cognitive side effects vary depending on how much time has passed after the last ECT session. Moreover, possible effects from the anesthetic dose on the mean charge delivered were not included in the analysis. There was no evaluation of subjective cognitive impairments after ECT. The extensive and potentially overwhelming neuropsychological testing may have influenced performance and missing data in depressed patients. Although focusing on less studied neuropsychological domains is highly relevant, it may be preferable to focus on fewer tests, including the VLMT delay task.

## 5 Conclusion

RUL-ECT was associated with significant autobiographical memory impairment in this study. The relationship between mean charge delivered and cognitive performance has been heterogeneous across different cognitive domains and requires further research. Significant effects of the mean charge delivered were found without a significant difference in cognitive functioning compared to a healthy control group, specifically for the VLMT delay score.

## Data Availability

Restrictions apply to the datasets: The datasets presented in this article are not readily available because local data protection laws. Requests to access the datasets should be directed to the University Hospital Bonn. Summary data will be made available by the authors, without undue reservation.
